# Best Practices and Innovative Solutions to Overcome Barriers to Delivering Policy, Systems and Environmental Changes in Rural Communities

**DOI:** 10.3390/nu10081012

**Published:** 2018-08-03

**Authors:** Lindsey Haynes-Maslow, Isabel Osborne, Stephanie B. Jilcott Pitts

**Affiliations:** 1 Department of Agricultural and Human Sciences, North Carolina State University, Raleigh, NC 27695, USA; 2 Department of Global Studies, University of North Carolina, Chapel Hill, NC 27514, USA; iosborne@live.unc.edu; 3 Department of Public Health, East Carolina University, Greenville, NC 27834, USA; jilcotts@ecu.edu

**Keywords:** rural populations, food assistance, low-income

## Abstract

To better understand the barriers to implementing policy; systems; and environmental (PSE) change initiatives within Supplemental Nutrition Assistance Program-Education (SNAP-Ed) programming in U.S. rural communities; as well as strategies to overcome these barriers, this study identifies: (1) the types of nutrition-related PSE SNAP-Ed programming currently being implemented in rural communities; (2) barriers to implementing PSE in rural communities; and (3) common best practices and innovative solutions to overcoming SNAP-Ed PSE implementation barriers. This mixed-methods study included online surveys and interviews across fifteen states. Participants were eligible if they: (1) were SNAP-Ed staff that were intimately aware of facilitators and barriers to implementing programs, (2) implemented at least 50% of their programming in rural communities, and (3) worked in their role for at least 12 months. Sixty-five staff completed the online survey and 27 participated in interviews. Barriers to PSE included obtaining community buy-in, the need for relationship building, and PSE education. Facilitators included finding community champions; identifying early “wins” so that community members could easily see PSE benefits. Partnerships between SNAP-Ed programs and non-SNAP-Ed organizations are essential to implementing PSE. SNAP-Ed staff should get buy-in from local leaders before implementing PSE. Technical assistance for rural SNAP-Ed programs would be helpful in promoting PSE.

## 1. Introduction

The prevalence of obesity in the United States (U.S.) creates a significant public health problem for both children and adults. In 2016, nearly 30% of children were overweight or obese and 30% of adults were obese [1]. Low-income individuals have the greatest risk of obesity, with 18% of children living below the federal poverty line being obese versus 10% of those living above the poverty level [2,3,4]. Geographically, nearly 16% of individuals in rural areas live below the poverty line, in contrast to 12% of individuals in urban areas [5]. Due to these income disparities, 16% of households in rural counties participate in the Supplemental Nutrition Assistance Program (SNAP) compared to 13% of household in urban counties [5]. Additionally, both children and adults living in rural areas are more likely to be obese than those living in urban areas [1,6,7]. These disparities in obesity prevalence may be partially due to disparities in access to healthy eating and physical activity opportunities. Compared to residents living in urban areas, residents in rural community have less access to healthy food and safe places to be active [8,9,10].

To help address food insecurity among low-income individuals in the U.S., the federal government created the SNAP in the 1960s [11]. Since its creation, SNAP has shifted its focus to also include improving dietary quality with the creation of federal nutrition education programs such as SNAP-Education (SNAP-Ed) [11]. The SNAP-Ed program was created in the 1980s as an optional cost-sharing federal program, to increase the likelihood that SNAP recipients make healthy food choices within a limited budget [11]. Individuals are eligible for SNAP-Ed if their incomes are at or below 185% of the Federal Poverty Level. SNAP-Ed reaches approximately 95 million low-income Americans in all 50 states, the District of Columbia, and three territories. Nationally, there are seven SNAP-Ed geographic regions. These regions include a range of states, and each state has at least one state SNAP-Ed implementing agency. Currently, there are 138 SNAP-Ed implementing agencies across the U.S. [11].

Prior to 2010, the majority of SNAP-Ed programming was traditional, direct- nutrition education (i.e., series-based classes or single session classes). Under traditional, direct-nutrition education, SNAP-Ed implementing agencies in rural areas faced barriers with recruiting families to attend sessions due to transportation, time, and lack of Internet access (for those living in mountainous regions) [12]. A 2004 national report concluded that more research was needed to determine whether SNAP-Ed behavior changes among low-income audiences was due to SNAP-Ed programs or other factors [13]. In 2010, the Healthy, Hunger-Free Kids Act added an additional component to SNAP-Ed encompassing broader policy, systems, and environmental (PSE) changes [11]. The Interpretive Guide to the SNAP-Ed Evaluation Framework defines a policy change as involving “a written plan or course of action designed to influence and determine decisions (includes the passing of laws, ordinances, resolutions, mandates, regulations or rules).” A system change “involves changes made to the rules or procedures within an organization—this can be a worksite, faith setting, school, non-profits, for-profit organizations.” An environmental change includes “changes made to the physical, social, or economic environment. SNAP-Ed interventions can be customized for different geographic regions, ages, and cultural settings” [14].

Utilizing PSE approaches to encourage healthy dietary changes can help overcome recruitment and attendance barriers, since PSE changes are typically meant to “make the healthy choice the easy choice” for a variety of populations. When PSE changes are implemented, a large segment of the SNAP-Ed population can be affected compared to a smaller segment of those who choose to enroll in traditional direction-education classes. However, SNAP-Ed implementing agencies vary widely in their ability and capacity to implement PSE interventions in their programs [15]. Two years after SNAP-Ed implementing agencies were encouraged to deliver PSE, nationally, approximately 98% were still focusing on direct education [16]. Furthermore, there have been prior studies that broadly covered rural PSE change implementation barriers, identifying barriers such as cultural differences, population size, and resulting limited human capital [17]. Strategies to overcome such barriers include building partnerships, and utilizing existing infrastructure [18,19]. There is also a great need to rigorously evaluate PSE in rural areas to determine effectiveness for future implementation [19,20]. Thus, more work is needed to learn how SNAP-Ed staff in rural areas view PSE, how they implement PSE, and how implementation and evaluation of PSE could be improved within the SNAP-Ed program, especially in rural areas.

Therefore, the purpose of this study was to better understand the barriers to implementing PSE approaches within SNAP-Ed programming in rural communities, as well as the strategies to overcome these barriers to SNAP-Ed programming in rural communities. In this study, we identified: (1) the types of nutrition-related PSE SNAP-Ed programming currently being implemented in rural communities, (2) barriers to implementing PSE in rural communities, and (3) common best practices and innovative solutions to overcoming SNAP-Ed PSE implementation barriers in rural communities. 

## 2. Materials and Methods 

To gain a better understand of the barriers to implementing SNAP-Ed PSE changes in rural communities, as well as the strategies to overcoming these barriers, we employed a mixed-method approach. We triangulated data obtained from an online survey administered to SNAP-Ed Staff across the United States, and subsequently conducted qualitative in-depth interviews among a sub-set of those SNAP-Ed staff who completed the online survey. Below, we outline additional details regarding methods of each data collection method.

### 2.1. Online Survey: Participant Recruitment and Analysis

We recruited staff at SNAP-Ed programs across the country to complete an online survey by using the Association for SNAP Education Nutrition Administration (ASNNA) and the CDC-funded Nutrition and Obesity Policy Research and Evaluation Network Rural Food Access Working Group (RFAWG) email listservs. The RFAWG includes academic researchers, public health and cooperative extension practitioners, and other experts focused on rural food access. Several RFAWG members connected us with SNAP-Ed implementing agency contacts in their respective states. The following were eligibility criteria to complete the survey and subsequent qualitative interview: (1) a SNAP-Ed staff member that was intimately aware of facilitators and barriers to implementing programs in the community; (2) individuals who have worked for a SNAP-Ed program that provides at least 50% of programming in rural communities; and worked in their role for at least 12 months.

This study was approved by North Carolina State University’s Institutional Review Board and all participants signified consent after being read an information sheet and agreeing to take part in the online survey and/or interview.

We used Qualtrics, an online survey program, to obtain information about PSE knowledge and experience, PSE activities, and partners engaged in PSE activities. Participants were asked to rate their level of knowledge with PSE approaches to behavior change using a 4-point Likert scale, ranging from “not knowledgeable at all” (=1) to “very knowledgeable” (=4). Participants were also asked to rate their level of experience with PSE using a 4-point Likert scale, ranging from “not experienced at all” to “very experienced”. Among those with experience implementing PSE, we asked in what types of community settings PSE initiatives were being implemented, with response options including community gardens, schools, farmers’ markets, healthy food retail and food pantries. We also asked what types of partners they were working with to implement those PSE initiatives, with response options including managers, principals, teachers, staff, food policy councils. SNAP-Ed staff could choose as many settings and partners that were applicable. Participants were asked about relevant outcome or evaluation data collected on PSE initiatives as an optional question. Data from the Qualtrics survey were exported from the program as a report, and simple descriptive statistics were calculated to determine frequencies of various responses.

### 2.2. In-Depth Interview Recruitment, Administration and Analysis

The last survey question asked study participants if they were interested in participating in an in-depth interview about barriers and facilitators to implementing SNAP-Ed within rural communities. Participants were asked to include their name and email and a member of the research team contacted them to schedule an interview. SNAP-Ed staff participated in telephone-administered interviews lasting 45–60 min using NoNotes, a professional telephone recording and audio sharing platform. Each interview was conducted by either Dr. Lindsey Haynes-Malsow (LHM) or Dr. Stephanie B. Jilcott Pitts (SJP) or using a semi-structured interview guide.

Participants were asked about their general experience with the SNAP-Ed program, such as how long they had worked with SNAP-Ed and in what capacity; what types of PSE initiatives in which their agency was currently engaged; what their favorite PSE initiative was and which initiative they felt was most effective; specific barriers to implementing PSE initiatives in rural communities; strategies that they used to overcome these barriers; future PSE initiatives that they were planning to implement in the following year, and how or if they were evaluating PSE initiatives.

Interviews were transcribed verbatim and analyzed using Atlas.ti version 7.0 (Scientific Software Development Gmbh, Berlin, Germany). LHM and SJP created a codebook based on independently coding a subset of three interviews. LHM and Isabel Osborne (IO) then independently applied these codes to all interview transcripts. Codes were reconciled and code memos, a 1–2 page summary based on each code, were written based on the all the codes. Final themes were compiled based on questions included on the interview guide. While participants were not asked specifically about “innovative” or “best” practices, LHM and SJP created an “innovative practice” code based on reviewing all PSE initiatives in accordance with SNAP-Ed guidelines, a description of how well the PSE initiative worked, and whether other participants had mentioned this practice. If the practice was mentioned only once, it was deemed “innovative”. If it was mentioned by more than one respondent, it was coded as a “best practice”.

## 3. Results

### 3.1. Online Survey

A total of 65 SNAP-Ed staff in rural implementing agencies completed online surveys. Sixteen (24.6%) responded they were somewhat knowledgeable, 26 (40.0%) knowledgeable, and 23 (35.4%) were very knowledgeable about PSE approaches to behavior change. However, when asked to rate their level of experience with PSE, 5 (7.8%) responded that they were not experienced at all, 25 (39.1%) were somewhat experienced, 23 (35.9%) were experienced, and 11 (17%) were very experienced. Table 1 shows that the top three settings in which respondents worked were schools (96%), food pantries (89%), and farmer’s markets (83%). The least frequently reported setting was supermarkets/supercenters, in which only 24% of respondents said they were implementing PSE initiatives. Among the “other” settings, 5 out of 11 respondents reported working in gardens. The type of partner varied by setting. For example, in schools, most SNAP-Ed staff partnered with the school staff such as principals or teachers. Surprisingly, those working in healthy food retail settings, such as corner stores and grocery stores, only reported working with the retail setting owner or manager as a partner approximately 25% of the time. Other partners they worked with in retail settings were health departments, food policy councils, and worksite staff (not owners or managers).

In terms of outcome or evaluation data collected on PSE initiatives (an optional question), responses varied greatly; however, some of the common responses were pounds of produce donated or sold; national SNAP-Ed indicators such as reach (the number of people that were in contact with the PSE initiative) and impact (behavior change such as fruit and vegetable intake). Among those that answered the optional question of whether they would be interested in participating in an interview 27 (41.5%) responded “yes” and 38 (58.5%) responded “no”.

### 3.2. In-Depth Interviews

**Figure 1:**
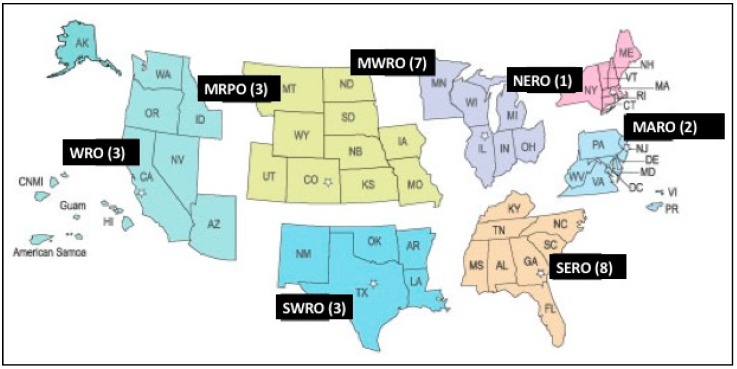
Interview Respondent Locations. Note: SERO (Southeast Regional Office); SWRO (Southwest Regional Office); MARO (Mid-Atlantic Regional Office); NERO (Northeast Regional Office); MWRO (Midwest Regional Office); MRPO (Mountain Regional Plains Office); WRO (West Regional Office)

A total of 27 interviews were conducted with SNAP-Ed staff in 15 states across all 7 SNAP-Ed regions (see Figure 1). The majority of interviewees had been working for SNAP-Ed for approximately 0–2 years (40.7%); were an average age of 39 years, female (81.5%), white/Caucasian (70.0%); conducted more than 75% of their work in rural communities (94.1%); contributed more than 75% of their time to PSE activities (37.0%); and less than 25% to direct-education (51.9%). See Table 2 for more information.

A total of 10 codes were created based on interviewees’ responses (see Table 3). These codes focused on PSE initiatives, barriers to implementing PSE, and facilitators to implementing PSE.

### 3.3. Types of Nutrition-Related SNAP-Ed PSE Initiatives Being implemented in Rural Communities

The most common PSE initiatives mentioned were gardens, school wellness-based initiatives, healthy food retail, farmers’ markets, and food pantries. In terms of implementing PSE initiatives in rural communities, the main challenges were funding, and level of PSE understanding among SNAP-Ed staff and stakeholders. However, strategies to overcome these challenges included working through partnerships and finding short-term PSE wins to demonstrate the importance of this approach to behavioral change. Below, we discuss the setting-based themes in more detail.

#### 3.3.1. Gardens

In the in-depth interviews, the most commonly mentioned PSE initiative was gardens. Many interviewees mentioned PSE initiatives related to gardening such as community gardens, school gardens, and personal gardens. These initiatives included activities such as teaching adults basic gardening skills, ways to increase garden viability such as bee keeping and composting, as well gardening education for students by showing them how and where fruits and vegetables grow, and generating general community interest in the value of gardening. A participant from SNAP-Ed Southeast Region commented: “I started a garden program at one of the schools, so we’re introducing the fourth grade students how to garden and where their food comes from because a lot of students, believe it or not, don’t know where their food comes from. They think it comes from Food Loin or Walmart. I’m tell them, ‘Well, technically yes, but it’s grown. It’s a process. Right now, I’m just at one school doing the garden program, but my goal is to eventually spread out and work with multiple grade levels, not just at the fourth grade levels, with that particular program.”

Setting-specific barriers—Gardens: Some SNAP-Ed staff mentioned barriers to implementing garden-based initiatives related to weather (late freezes, extreme heat, and droughts) or when working in schools, having to compete for time with schools’ teaching requirements and curriculum standards.

Setting-specific best practices—Gardens: Several interviewees mentioned potential facilitators to increase school gardening PSE programs, such as encouraging all schools to have a garden (making it the cultural norm) and full support from the community.

Setting-specific innovative solutions—Gardens: One innovative solution mentioned was having work meetings in gardens. As a participant from SNAP-Ed Mountains Plains Region said, “Our educator partnered with Extension and they helped tell her (worksite owner) what kind of plants grow well there and taught her about companion plants and that kind of thing. And she partnered with other agencies so they could have working meetings and actually work in the garden while they were discussing business.” This increased both awareness of the garden and helped build a better partnership between the SNAP-Ed staff and worksite.

#### 3.3.2. School Wellness Initiatives

Many of the interviewees described effective PSE strategies within schools in their rural communities. These strategies included smarter lunchrooms, farm-to-school projects, school gardens, and taste tastings. In addition, many SNAP-Ed staff encouraged schools to connect direct-education to PSE initiatives so could reinforce lessons learned in class and maximize exposure to healthy living initiatives. As a participant from SNAP-Ed Western Region said, “I would have to say that for all our counties, our PSE work is primarily in the school setting. We do edible school gardens, local school wellness policy participation, school stenciling projects and mural projects, physical activity integration, technical and training assistance for teachers, and, Smarter Lunchroom Movement activities.”

Setting Specific Barriers—School-Wellness: Several SNAP-Ed staff discussed competing for class time as a barrier to implementing PSE-based programs. Since some regional elementary schools have end-of-year grade exams, schools are fairly focused on ensuring that students are well prepared for these exams; time that is taken away from teaching for this test is seen as a competing interest. Additionally, not having buy-in from school principals or teachers was also a barrier for newer SNAP-Ed staff.

Setting-specific best practices—School Wellness: To increase school interest in SNAP-Ed programming, some SNAP-Ed staff explained how they created programs that did not require teacher participation. Some staff explained that if teachers were required to help with programming, they would find it burdensome on an already heavy teaching load. Other SNAP-Ed staff let school leadership choose which PSE initiative(s) they wanted to implement. Generally, schools with PSE initiatives were more likely to have community partners helping them sustain their activities, such as donating school garden produce to food pantries.

Setting-specific innovative solution—School-Wellness Based: One innovative practice used at a school involved converting an old and outdated pool into a garden and converting the pool house for produce processing, and tool and lumber storage. As a participant from SNAP-Ed Northeast Region stated about the initiative, “It’s been very successful…and after all this time finally getting produce into the cafeteria.”

#### 3.3.3. Healthy Food Retail

Many SNAP-Ed staff discussed healthy food retail in the context of working with grocery stores and corner stores. These initiatives included posting new signage to direct people towards healthier food items, graphically designing advertisements for healthy food specials, deals, and sales, and creating healthy food check-out lines where produce and other healthy “grab and go” snacks were easily accessible. Some healthy food retail projects were well-received in the community, as one participant in the SNAP-Ed Northeast Region said their “corner store makeover” was extremely popular among community members.

Setting Specific Barriers—Healthy Food Retail: When working on healthy food retail, some SNAP-Ed staff experienced lack of buy-in from store owners—as they did not want to change their food inventory and what they sold to customers. Additionally, several SNAP-Ed staff had difficulty measuring the impact of their healthy food retail initiatives to determine how effective they were, as well as ensuring that store owners and managers continued to post signage and stock fresh produce. 

Setting-specific best practices—Healthy Food Retail: To help overcome lack of buy-in, many SNAP-Ed staff described ensuring that they communicated regularly with the store owners and discussing with stores the benefits of how these small, no or low cost changes could improve the community’s diet and health. One SNAP-Ed staff member from Southeast Region summarized their experience as, “Just getting my foot in the door with the corner store was very difficult because the owner wasn’t on-board at first, which I can understand because of the community and the dynamics of it. His store was known for hot dogs and ice cream—and getting him to implement change in the store did not come easy for his customers. He was trying to think on that aspect, but then as I continued to work and talk with him, he was open to the idea of stocking fruits. He started to put the fruits out and just to see what would happen, and on the first day he did it, he said he had to stock up twice.”

Setting-specific innovative solutions—Healthy Food Retail: One SNAP-Ed staff member encouraged bundling (combining multiple food products and selling them together) healthy foods with other high demand foods. Essentially, making the bundled price (the high demand food item plus healthy snacks) cheaper than the price of a high demand food item plus an unhealthy snack. Additionally, another SNAP-Ed staff member was working with an independent grocery store owner to develop a mobile grocery store to increase access to healthy food in areas with limited access. As the SNAP-Ed interviewee from the Southeast Region stated, “We’re really trying to work on every level to help with this opportunity, help with the funding, and help our educators. We’ve got just a stellar independent grocer here locally. And thank goodness for me he’s local so I can work directly with him and we can work out models and look at the economics around the model and things of that nature. And he actually is—they’re about to pilot starting in the first quarter of 2018 online SNAP acceptance.”

#### 3.3.4. Farmer’s Markets

SNAP-Ed staff commented on working with their community farmer’s markets using PSE approaches or how they had plans to improve it in the future. Many SNAP-Ed staff worked on encouraging farmer’s markets to accept SNAP as part of their PSE initiative. SNAP-Ed staff would also provide food tastings and cooking demonstrations at farmer’s markets, and provide hand-outs with recipes or health tips. Additionally, SNAP-Ed staff collaborated with other organizations to help work with farmer’s markets.

Setting Specific Barriers—Farmer’s Markets: Some communities did not have farmer’s markets, so SNAP-Ed staff worked on trying to create one. However, this process is time consuming, expensive, and can be logistically challenging as many farmer’s markets are usually not located in areas that low-income individuals frequent. Additionally, even if a rural community did have a farmer’s market, they lacked SNAP, which was seen as a major barrier for low-income customers.

Setting-specific best practices—Farmer’s Markets: Several SNAP-Ed staff talked about partnering with other organizations to implement incentive programs. As one SNAP-Ed staff from the Mountains Plains Region explained, “We worked to encourage them (the farmer’s market) to do incentive programs, so if people do redeem SNAP benefits they may get ‘double up bucks’, they may get extra matching funds for whatever they redeem with their SNAP benefits, or they might get like an extra basket of fruits and vegetables in addition to what they would go buy.”

Setting-specific innovative solution—Farmer’s Markets: One farmer’s market PSE initiative involved a policy practice. “One of the PSE changes that we did put in place at our farmer’s market was demonstrating healthy recipes utilizing some of the fruits and vegetables that were being featured there that week, so anybody that came in and did a food demonstration had to serve fruits and vegetables. It had to be healthy. Water is served as the beverage of choice there, whereas before they may have been doing punch or something like that,” said a participant from SNAP-Ed Southeast Region.

#### 3.3.5. Food Pantries

Many interviewees commented on the prevalence of working with food pantries as part of their PSE programming. The majority of work with food pantries was in collaboration with others organizations or projects—building on behavioral economic movements such as nudging customers towards healthier foods [13]. Food pantries often offered direct food to pantry clients, and SNAP-Ed staff discussed coming into the pantry and using a client choice model, where food pantry customers could choose their food contents—but they were encouraged to choose healthy options. One SNAP-Ed agent from the Southeast Region described a specific example of this: “This particular pantry, there’s a box that’s pre-made—it has meat, some pasta, and canned goods. They get a box of these staple food items, but then they get to choose their produce items afterwards. It’s a little bit of a client choice model, but not entirely. There are other pantries that allow you to choose every single food item that you want. That way, you don’t end up with food that’s unhealthy if you don’t want it. The clients have a choice in that. This one is kind of a half client choice, but they still get a pre-made box.”

Setting-specific barriers—Food Pantries: Several SNAP-Ed staff discussed the lack of buy-in from food pantry owners—as they did not want to change food distributions policy. Even when SNAP-Ed staff did get food pantry owners to try behavioral economics, they had to check-in frequently to ensure that the food pantry director or coordinator continued to use behavioral economics to encourage clients to choose healthy foods.

Setting-specific best practices—Food Pantries: SNAP-Ed staff discussed working with food pantry owners and managers and moving towards providing healthier food options within the pantries.

Setting-specific innovative solution—Food Pantries: One SNAP-Ed staff explained partnering with a medium-security prison on their produce-growing contest—prisons try to grow the most produce and after the contest, the produce is donated across the community. While SNAP-Ed funding does not allow funds to be spent within the prison population, SNAP-Ed staff members were initially invited by the warden to encourage volunteers to help find places to distribute the produce. As a SNAP-Ed staff from the Midwest Region explained, “All of a sudden, we have this huge group of volunteers that wants to help distribute 12,000 pounds of produce, because one or two people can’t distribute all of that. So all of a sudden, we have all of these volunteers who are on-board to help take all the produce to a central location, and then they’ll take it to these food pantries or these programs that are low income. Our summer day camp that the YMCA runs is like 75% free and reduced lunch, so they’ll get some, and distribute it to places that (A) don’t have a budget to buy it, but (B) desperately need it for the clients that they’re serving. I think that one of the biggest things with communities is just having the support of the people who live there, play there, work there.”

### 3.4. Barriers to Implementing SNAP-Ed PSE in Rural Communities

There were three general barriers participants discussed when implementing SNAP-Ed PSE initiatives in rural communities: funding, lack of infrastructure, and lack of PSE understanding. 

#### 3.4.1. Funding

In terms of funding, most participants discussed the lack of funding available for PSE initiatives. As one SNAP-Ed Midwest Region participant said, “The biggest barrier is funding because lot of people like these (PSE) ideas, but there’s very little extra money laying around”. Additionally, due to SNAP-Ed allowable and unallowable costs, as defined by the U.S. Department of Agriculture (USDA), grantees cannot use SNAP-Ed funds for infrastructure (such as building sidewalks or playground) or to purchase food (such as funding “double up bucks” program).

#### 3.4.2. Lack of PSE Understanding

Even though PSE has been a part of SNAP-Ed programming for several years now, there is still a lack of PSE understanding among some SNAP-Ed staff and stakeholders. Several interviewees discussed SNAP-Ed staff and stakeholders (community members, local leaders) as not having as high a regard for PSE as traditional direct-education. SNAP-Ed staff explained that some stakeholders wondered why PSE initiatives were important and questioned whether they would truly make an impact. Other SNAP-Ed staff and stakeholders misinterpreted the definition of policy, “Even though I’m comfortable with PSE, many of our SNAP-Ed assistants are not. ‘Policy’ is a scary word to them and I think a training to make them feel more comfortable about it, and realize that it’s not always about talking to senators, would be helpful”. (Participant from the SNAP-Ed Mid-Atlantic Region)

### 3.5. Best Practices’ to Overcoming SNAP-Ed PSE Implementation Barriers in Rural Communities

While some barriers to SNAP-Ed programming cannot be overcome with SNAP-Ed grant funding, interviewees discussed strategies they have used to overcome challenges.

#### 3.5.1. Overarching Best Practice: Partnerships

The most frequently cited facilitator to implementing SNAP-Ed programming was partnering with other community initiatives or organizations, building relationships with coalitions, wellness committees, advisory groups, or food policy councils. Partnerships were often useful with SNAP-Ed, as funds could not be used to cover the cost of a certain project, such as renovating a walking trail, but another organization could. Additionally, being in a rural community was seen as an advantage since, “everybody knows each other, so they have the ability to network and build or enhance partnerships”—(participant from the SNAP-Ed Midwest Region). Partnerships were also seen as a way to ensure sustainability of projects—especially beyond SNAP-Ed funding periods. Another interviewee from the SNAP-Ed Midwest Region stated, “SNAP-Ed is an important piece of what people are doing, but we don’t want them to do it in a silo, and we want to have community buy-in to advance PSE strategies, right? So working at that broader level with multisector representatives really helps set those changes up not only for adoption but also for sustainability in the future”.

#### 3.5.2. Overarching Best Practice: Communicating Short-Term PSE Wins in the Community

Due to the fact that some SNAP-Ed staff and stakeholders did not understand the definition or value of PSE, many interviewees talked about the need for “short-term PSE wins” to demonstrate how impactful it could be in the community. Recognizing that SNAP-Ed funding is renewed every year and that PSE can take years to implement and achieve desired results, many SNAP-Ed staff talked about finding PSE work that was already underway in the community and assisting with those projects. One participant from the SNAP-Ed Midwest Region discussed helping with a complete street initiative and promoting connector trails to a major walking trail in the community: “I think a really helpful thing is to find a way to partner with and plug into things that are already sort of ‘easy wins’ that are already starting or initiated in some way and then trying to help shape them and direct them to the more healthy direction.” Visually demonstrating to the staff, community, and other stakeholders that PSE can be an extremely influential and impactful strategy for behavior change is key to local buy-in and longer term success.

## 4. Discussion

The results of this study provide a deeper understanding of the types of nutrition-related SNAP-Ed PSE initiatives being implemented currently in rural communities, implementation barriers, and both “best practice” strategies and innovative solutions to overcoming SNAP-Ed PSE implementation barriers in rural communities.

Based on online surveys and interviews, we found that SNAP-Ed PSE initiatives in rural areas included working with schools, gardens, food pantries, farmers’ markets, and food retail settings like corner stores and supermarkets. Nearly 96% of the participants reported working with schools. Another study conducted with 15 public health practitioners about environmental and policy changes to support healthy eating and physical activity in rural communities also identified schools as a main setting to implement initiatives [17].

Interviewees offered a variety of creative solutions to overcome barriers, including worksite meetings in gardens, repurposing an old pool, and working with an independent grocer on mobile markets. Such creativity and partnering with appropriate organizations and stakeholders are two vital components to overcome challenges to PSE in rural areas. Building partnerships and having buy-in from local leaders, such as the city council, was also seen as an important strategy in implementing environmental and policy interventions to support physical activity and healthy eating in rural communities, in another study [17].

One major barrier included funding restrictions due to SNAP-Ed allowable and unallowable costs. The barrier of funding restrictions was often overcome by partnering with other community groups, foundations, or grantees. Partnerships were noted in prior studies [18,19] as important solutions to PSE implementation barriers, and were a key best practice in the current study. Barnidge et al. found that the most important partners for implementing physical activity and healthy eating in rural communities were city councils or mayors, local businesses, schools, health promotion organizations, healthcare facilities or healthcare providers [17]. Similarly, partners mentioned in the current study included school staff, food policy council members, health department staff, and worksite staff.

The goal of PSE strategies is to make a healthy choice, an easy choice, or even the default choice. This quality of PSE makes it appealing, as it does not require attendance from SNAP-Ed participants, overcoming commonly cited transportation and time barriers [15]. In rural communities, PSE could have greater reach and impact due to transportation and infrastructure barriers that these communities face. In a recent study, researchers found that socioeconomic status, rather than geographic location, was a better predicator of transportation barriers. In a systematic literature review across 25 studies, between 10–51% of lower-income individuals reported transportation as a barrier [21]. In terms of infrastructure as barrier to health living, one study among eight southeastern rural communities in the U.S. found that rural communities lack policies and programs to support safe places to be active, especially sidewalks or policies related to schools, allowing the community to use their facilities outside of school hours (such as shared use policies) [22]. Additionally, access to healthy foods in rural communities is limited, as residents have greater exposure to retail food outlets that sell a limited selection of healthy food items [23,24,25,26]. Fortunately, research has suggested that healthy retail initiatives in small food stores, which can be supported by SNAP-Ed, can be a strategy to improve the quality of food in stores [27,28].

As mentioned in the interviews, some SNAP-Ed staff have varying levels of understanding PSE and some stakeholders do not fully comprehend PSE. A solution that others have recommended is to not use the words, “policy, systems, or environmental” change when talking with other community members, organizational leaders, and other stakeholders. Our study showed that these terms were perceived as very top-down, high-level, and abstract—all of which were not viewed as favorable among rural community members.

One way to demonstrate the impact of PSE on behavior change is through rigorous impact and outcome evaluation. Demonstrating that a policy, system, or environmental change can actually transform the environment and result in more positive and healthy behaviors is critical to indicating the utility of PSE for various stakeholder groups and can provide evidence of a “short term win”. For example, a SNAP-Ed program assisted a local non-profit with renovation of a trail by providing signage and promoting the trail opening. However, the majority of SNAP-Ed interviewees expressed difficulties in evaluating PSE and evaluation methods as settings varied greatly across the different SNAP-Ed programs. Others have called for more rigorous evaluation of PSE in rural communities [18]. While the SNAP-Ed Evaluation Framework and Interpretive Guide offers many examples and suggestions on how to collect data, one barrier to evaluating programs is the lack of funding and time for SNAP-Ed programs to rigorously evaluate PSE initiatives [14]. SNAP-Ed implementers could have successful partnerships with evaluators of neighboring research institutes or universities to conduct rigorous evaluation of PSE initiatives.

## 5. Conclusions

The goal of SNAP-Ed is to help improve the chances of lower-income children and families making healthy food choices within a limited budget. Using a multi-level approach by combining direct education with PSE efforts gives SNAP-Ed the opportunity to facilitate positive behavior change. However, this requires that SNAP-Ed implementing agencies be knowledgeable and comfortable with delivering both types of programming [15]. The current study illustrates barriers, and creative solutions that other rural SNAP Ed staff can implement, to increase the success and sustainability of rural PSE initiatives.

This study has several strengths and limitations. First, it included SNAP-Ed staff from all seven SNAP-Ed regions. Additionally, this study is strengthened by having two trained qualitative researchers conduct all interviews, double-code transcripts, and reconcile all codes. Some of the limitations are the potential selection bias for those who chose to participate in the interviews. Since the online survey asked many questions about PSE, SNAP-Ed staff with more experience and interest in implementing PSE might have volunteered for an interview, rather than those with less experience or interest. 

Few studies have investigated implementing SNAP-Ed PSE initiatives in rural communities [22]. This study revealed innovative strategies and best practices for implementing PSE in rural areas, which can assist similar SNAP-Ed implementing agencies working in rural communities to overcome barriers to PSE. The ultimate goal of implementing SNAP-Ed PSE initiatives is to empower low-income individuals to make healthy choices within their homes, schools, worksites, and communities. Future studies should focus on disseminating innovative strategies and best practices for implementing PSE in rural communities and evaluate whether these strategies are effective at positively changing health behaviors.

## Figures and Tables

**Table 1:** PSE Settings and Partners.

Where SNAP-Ed PSE Programming is Located		Partner				
Settting	I Work in This Setting	Health Departments	Retail Food Store Owner	Food Policy Councils	Worksite Staff	Other
Childcare center	61%	11%	0%	4%	30%	18%
School	96%	7%	5%	11%	50%	23%
Workplace	64%	12%	2%	9%	19%	22%
Senior Center	68%	15%	0%	7%	22%	25%
Faith-based locations	66%	5%	2%	12%	20%	27%
Corner store	54%	11%	22%	3%	6%	12%
Grocery store	53%	7%	26%	2%	4%	14%
Supermarket/supercenter	24%	2%	10%	2%	4%	6%
Food Pantry	89%	15%	4%	17%	27%	27%
Farmer’s Market	83%	13%	3%	13%	22%	32%
Other Setting	44%	2%	0%	2%	12%	18%

**Table 2:** Interview Participant Demographics (*n* = 27).

Characteristic	Number	Percent
Number of years working for SNAP-Ed		
0–2	11	40.7%
3–5	6	22.2%
6–10	3	11.1%
>10	5	18.5%
Mean Age (years)		39
Gender		
Male	2	7.4%
Female	22	81.5%
Race/Ethnicity		
White	19	70.0%
Native American/American Indian	1	3.7%
Hispanic or Latino	2	7.4%
African American or Black	2	7.4%
Work Conducted in Rural Communities		
25–50%	5	18.5%
51–75%	4	14.8%
>75%	16	94.1%
PSE Work		
<25%	6	22.2%
25–50%	5	18.5%
51–75%	4	14.8%
>75%	10	37.0%
Direct-Ed Work		
<25%	14	51.9%
25–50%	4	14.8%
51–75%	4	14.8%
>75%	3	11.1%

**Table 3:** Codebook for Interviews.

Code	Definition	Illustrative Quote	Frequency
PSE Initiative: Garden-based	Mention of garden-based PSE strategies	“The biggest thing is that I have worked with a sort of small coalition of people in the community to start a new community garden park, we found a big three-acre parcel of land and then got a garden park sort of design put together that includes garden plots and a playground and a walking trail and a pavilion and a hoop house and all kinds of things that make it both a place where people can exercise and come and gather as a community, and then also grow food for themselves in these plots that are free.”	27
PSE Initiative: School Wellness-Based	Mention of school-wellness PSE strategies	“We’ve worked on developing a training module for the staff and teachers to learn more about the school wellness policy. The school system has had a wellness policy for a number of years and it is a pretty good wellness policy, but we did a survey last year and found out most of the staff don’t really know anything about it, so the idea is that they have to complete these online little video modules every year before the start of the school year.”	21
PSE Initiative: Healthy Food Retail	Mention of healthy food retail PSE strategies, such as corner stores, grocery stores, supermarkets	“There is a PSE in the region that’s a corner store makeover, which it’s super popular around the country, so I’m sure you’ve heard of that one.”	21
PSE Initiative: Farmers’ Market	Mention of farmers’ market PSE strategies	“We work in farmers markets doing food demonstrations and recipe kind of hand outs and other direct education, kind of very brief handouts.”	13
PSE Initiative: Food Pantries	Mention of food pantry PSE strategies	“I’ve partnered our regional food bank, and we are working on a nudging pilot. SNAP-Ed has partnered with them to provide signage. We’re offering volunteer education. I’ve done food demos and recipe cards for produce items that they know they’ll have excess of.”	12
Lack of healthy food and physical activity Infrastructure	There is lack of infrastructure in rural communities that make access to healthy food and physical activity difficult for the population.	“Somebody in a rural area might have to drive like a half hour or an hour to go get groceries that doesn’t have anything fresh.”	59
Partnerships	Partnering with other community initiatives or organizations, building relationships with partners, coalitions, wellness committees, advisory groups, ect.	“One of my favorite PSE strategies, is really having SNAP-Ed partners who are connecting with existing opportunities in their communities and regions to bring the SNAP-Ed lens to these coalitions that have some kind of health focus and then identifying strategies that allows those multi-sector partners around the table to leverage resources and work to advance PSE work that some of that’s SNAP-Ed, but it also goes beyond SNAP-Ed.”	65
Short-term PSE wins	Recognizing the importance of having short term wins to prove that PSE can be an effective strategy for behavior change, this includes being intentional where you work choosing locations where you think your programming will be successful.	“Over the past couple of years, we have been required to look at that environmental level and think about the short-term piece, so your partnerships, needs and readiness, that kind of stuff.”	11
Level of understanding of PSEs	Mentions the lack of understanding of PSE as a barrier to implementation	“We’re definitely going to start with a statewide training so that everybody understands the importance of PSE, and when I say everybody, we’re talking about not just people who are health educators. We’re trying to be pretty strategic in who we invite to the training and who we invite as a coach. We’re talking about our parks department, our state, and city, and town planners, our faith-based leaders, trying to really be all-inclusive of everybody who impacts health to really help folks understand that everybody has an impact on health.”	33
Funding	Lack of funding for the amount of work that needs to be done; also includes SNAP-Ed’s lack of ability to cover incentives for participants	“I would like to have the time or staffing to go out and really spend time with our educators and their partners developing relationships where our partners understood the importance of PSE work. I need them to do direct education and PSE work and get their reporting done on time;, I just don’t have a lot of time left for them. And so I would say I would just need a bigger chunk of money so I could have more people to really dive in.”	52

## References

[1] Lundeen E.A., Park S., Pan L., O’Toole T., Matthews K., Blanck H.M. (2018). Obesity Prevalence Among Adults Living in Metropolitan and Nonmetropolitan Counties—United States, 2016. MMWR.

[2] Ogden C.L., Carroll M.D., Kit B.K., Flegal K.M. (2014). Prevalence of childhood and adult obesity in the United States, 2011–2012. JAMA.

[3] Singh G.K., Siahpush M., Kogan M.D. (2010). Rising social inequalities in U.S. childhood obesity, 2003–2007. Am. Educ. J..

[4] Skelton J.A., Cook S.R., Auinger P., Klein J.D., Barlow S.E. (2009). Prevalence and trends of severe obesity among U.S. children and adolescents. Acad. Peds..

[5] Food Research Action Center Participation in the Supplemental Nutrition Assistance Program (SNAP) Highest in Rural Areas and Small Towns, New Data Tool Reveals. http://frac.org/news/participation-supplemental-nutrition-assistance-program-snap-highest-rural-areas-small-towns-new-data-tool-reveals.

[6] Davis A.M., Bennett K.J., Befort C., Nollen N. (2011). Obesity and related health behaviors among urban and rural children in the United States: Data from the National Health and Nutrition Examination Survey 2003–2004 and 2005–2006. J. Ped. Psychol..

[7] Johnson A., Mohamdi A. (2015). Urban-rural differences in childhood and adolescent obesity in the United States: A systematic revie and meta-analysis. Child. Obes..

[8] Murimi M.W., Harpel T. (2010). Practicing preventive health: the underlying culture among low-income rural populations. J. Rural Health.

[9] Hennessy E., Kraak V.I., Hyatt R.R., Bloom J., Fenton M., Wagoner C., Economos C.D. (2010). Active living for rural children: Community perspectives using PhotoVOICE. Am. J. Prev. Med..

[10] Moore J.B., Jilcott S.B., Shores K.A., Evenson K.R., Brownson R.C., Novick L.F. (2010). A qualitative examination of perceived barriers and facilitators of physical activity for urban and rural youth. Health Educ. Res..

[11] U.S. Department of Agriculture (USDA) Supplemental Nutrition Assistance Program Education (SNAP-Ed). www.fns.usda.gov/snap/supplemental-nutrition-education-assistance-program-education-snap-ed.

[12] Leung C.W., Hoffnagle E.E., Lindsay A.C., Lofink H.E., Hoffman V.A., Turrell S., Willett W.C., Blumenthal S.J. (2013). A Qualitative Study of Diverse Experts’ Views about Barriers and Strategies to Improve the Diets and Health of Supplemental Nutrition Assistance Program (SNAP) Beneficiaries. JAND.

[13] United States General Accounting Office (2004). Nutrition Education: USDA Provides Services through Multiple Programs, but Stronger Linkage among Efforts Are Needed. http://www.gao.gov/new.items/d04528.

[14] U.S. Department of Agriculture (USDA) Interpretive Guide to the SNAP-Ed Evaluation Framework. https://snaped.fns.usda.gov/evaluation/evaluation-framework-and-interpretive-guide.

[15] Frank K. (2016). Delphi Study Summary: Barriers, Facilitators, and Training Needs for Successful PSE Implementation in SNAP-Ed and EFNEP. http://snapedpse.org/resources/RNECE-PSE%20Delphi%20summary%202016.

[16] United States Department of Agriculture (USDA) Food Stamp Nutrition Education Systems Review: Final Report. http://www.fns.usda.gov/ora/MENU/Published/NutritionEducation/Files/FSNESystemsReviewExecSummary.

[17] Barnidge E.K. (2013). Understanding and addressing barriers to implementation of environmental and policy interventions to support physical activity and healthy eating in rural communities. J. Rural Health.

[18] Calancie L., Leeman J., Pitts S.B., Khan L.K., Fleischhacker S., Evenson K.R., Schreiner M., Byker C., Owens C., McGuirt J. (2015). Nutrition-Related Policy and Environmental Strategies to Prevent Obesity in Rural Communities: A Systematic Review of the Literature, 2002–2013. Prev. Chron. Dis..

[19] Wilson N.L., Just D.R., Swigert J., Wansink B. (2016). Food pantry selection solutions: A randomized controlled trial in client-choice food pantries to nudge clients to targeted foods. J. Public Health.

[20] Wyker B.A., Jordan P., Quigley D.L. (2012). Evaluation of supplemental nutrition assistance program education: application of behavioral theory and survey validation. JNEB.

[21] Syed S.T., Gerber B.S., Sharp L.K. (2013). Traveling towards disease: Transportation barriers to health care access. J. Community Health.

[22] Robinson J.C., Carson T.L., Johnson E.R., Hardy C.M., Shikany J.M., Green E., Willis L.M., Marron J.V., Li Y., Lee C.H. (2014). Assessing environmental support for better health: Active living opportunity audits in rural communities in the southern United States. Prev. Med..

[23] Jilcott S.B., Liu H., Moore J.B., Bethel J., Wilson J., Ammerman A.S. (2010). Commute times, food retail gaps, and weight status in rural and urban North Carolina counties. Prev. Chron. Dis..

[24] D’Angelo H., Ammerman A., Gordon-Larsen P., Linnan L., Lytle L., Ribisl K. (2016). Small food store retailers’ willingness to implement healthy store strategies in rural North Carolina. J. Community Health.

[25] McGuirt J.T., Pitts S.B.J., Ammerman A., Prelip M., Hillstrom K., Garcia R.E., McCarthy W.J. (2015). A mixed methods comparison of urban and rural retail corner stores. AIMS Public Health.

[26] Pinard C.A., Shanks C.B., Harden S.M., Yaroch A.L. (2016). An integrative literature review of small food store research across urban and rural communities in the US. Prev. Med. Rep..

[27] Gittelsohn J. (2012). Interventions in small food stores to change the food environment, improve diet, and reduce risk of chronic disease. Prev. Chron. Dis..

[28] Langellier B.A., Garza J.R., Prelip M.L., Glik D., Brookmeyer R., Ortega A.N. (2013). Corner Store Inventories, Purchases, and Strategies for Intervention: A Review of the Literature. Calif. J. Health. Promot..

